# Supplementary material for: Developing a cosmetic formulation containing lipase produced by the fungus Aspergillus terreus
Source: PLoS One. 2025 May 7;20(5):e0322106. doi: 10.1371/journal.pone.0322106 (PMC12058140; doi:10.1371/journal.pone.0322106)
Supplement: Attachment — Submitted filename: Response to Reviewers.docx [file pone.0322106.s005.docx]

**RESPONSE TO REVIEWERS**

**Journal requirements:**

1. When submitting your revision, we need you to address these additional requirements. Please ensure that your manuscript meets PLOS ONE's style requirements, including those for file naming. The PLOS ONE style templates can be found at https://journals.plos.org/plosone/s/file?id=wjVg/PLOSOne_formatting_sample_main_body.pdf and <https://journals.plos.org/plosone/s/file?id=ba62/PLOSOne_formatting_sample_title_authors_affiliations.pdf>.

*We appreciate the comments. The suggestion was accepted*

2. We note that the grant information you provided in the ‘Funding Information’ and ‘Financial Disclosure’ sections do not match. When you resubmit, please ensure that you provide the correct grant numbers for the awards you received for your study in the ‘Funding Information’ section.

*This study was partly financed by the Coordenação de Aperfeiçoamento de Pessoal de Nível Superior - Brasil (CAPES) - Finance Code 001.*

3. Thank you for stating the following financial disclosure: This work received financial support from the Coordenação de Aperfeiçoamento de Pessoal de Nível Superior, which granted the scholarship (001).]. Please state what role the funders took in the study.  If the funders had no role, please state: "The funders had no role in study design, data collection and analysis, decision to publish, or preparation of the manuscript. If this statement is not correct you must amend it as needed. Please include this amended Role of Funder statement in your cover letter; we will change the online submission form on your behalf.

*The funders had no role in study design, data collection and analysis, decision to publish, or preparation of the manuscript.*

4. We note that figure 2 in your submission contain copyrighted images. All PLOS content is published under the Creative Commons Attribution License (CC BY 4.0), which means that the manuscript, images, and Supporting Information files will be freely available online, and any third party is permitted to access, download, copy, distribute, and use these materials in any way, even commercially, with proper attribution. For more information, see our copyright guidelines: <http://journals.plos.org/plosone/s/licenses-and-copyright>. We require you to either (1) present written permission from the copyright holder to publish these figures specifically under the CC BY 4.0 license, or (2) remove the figures from your submission: a. You may seek permission from the original copyright holder of Figure 2 to publish the content specifically under the CC BY 4.0 license.

We recommend that you contact the original copyright holder with the Content Permission Form (http://journals.plos.org/plosone/s/file?id=7c09/content-permission-form.pdf) and the following text:

“I request permission for the open-access journal PLOS ONE to publish XXX under the Creative Commons Attribution License (CCAL) CC BY 4.0 (http://creativecommons.org/licenses/by/4.0/). Please be aware that this license allows unrestricted use and distribution, even commercially, by third parties. Please reply and provide explicit written permission to publish XXX under a CC BY license and complete the attached form.”

*Figure 2 does not contain copyrighted images. Figure 2 contains Scanning Electron Microscopy images obtained by the Laboratory of Structural Characterization of Materials, Department of Materials Engineering – DEMat, Federal University of Rio Grande do Norte. The captions of Figure 2 have been removed not to generate doubts regarding copyright.*

Reviewer #1:The paper aimed to evaluate the potential of lipase from *Aspergillus terreus* (active ingredient) in cosmetic formulations. The enzymes were characterized using several instrumental techniques. Reviewing the paper I found several drawbacks and therefore I must say that the paper is not suitable for publication in Plos One. Introduction section is poorly written and should be a little bit elaborative which will include scientific interests to the readers. In spite of sufficient data, instrumental techniques has not been well described/justified. Conclusion section is poorly written. Therefore I must reject it.

*Thank you for considering our manuscript. We appreciate the comments and made careful reorganization of the content, considering all the points mentioned. We made the proper changes in the text highlighting in yellow*
